# A Bio-Inspired Data-Driven Locomotion Optimization Framework for Adaptive Soft Inchworm Robots

**DOI:** 10.3390/biomimetics10050325

**Published:** 2025-05-16

**Authors:** Mahtab Behzadfar, Arsalan Karimpourfard, Yue Feng

**Affiliations:** 1 School of Mechatronical Engineering, Beijing Institute of Technology, Beijing 100081, China; mahtabbehzadfar@bit.edu.cn; 2 Department of Mechanical Engineering, Amirkabir University of Technology, Tehran 15914-35111, Iran; karimpour.arsalan@gmail.com

**Keywords:** soft robotics, bio-inspired locomotion, energy efficiency, neural network modeling, particle swarm optimization, locomotion optimization, bio-inspired design

## Abstract

This paper presents a data-driven framework for optimizing energy-efficient locomotion in a bio-inspired soft inchworm robot. Leveraging a feedforward neural network, the proposed approach accurately models the nonlinear relationships between actuation parameters (pressure, frequency) and environmental conditions (surface friction). The neural network achieves superior velocity prediction performance, with a coefficient of determination (R^2^) of 0.9362 and a root mean squared error (RMSE) of 0.3898, surpassing previously reported models, including linear regression, LASSO, decision trees, and random forests. Particle Swarm Optimization (PSO) is integrated to maximize locomotion efficiency by optimizing the velocity-to-pressure ratio and adaptively minimizing input pressure for target velocities across diverse terrains. Experimental results demonstrate that the framework achieves an average 9.88% reduction in required pressure for efficient movement and a 6.45% reduction for stable locomotion, with the neural network enabling robust adaptation to varying surfaces. This dual optimization strategy ensures both energy savings and adaptive performance, advancing the deployment of soft robots in diverse environments.

## 1. Introduction

Soft robotics is an innovative field that takes inspiration from biological organisms such as octopuses, snakes, and inchworms to create robots with flexible, deformable bodies capable of adaptive movement and manipulation in unpredictable environments. By utilizing soft materials, these robots can safely interact with delicate objects and navigate complex terrains where traditional rigid robots are limited. The integration of machine learning (ML) and deep learning (DL) has significantly advanced the capabilities of soft robots, enabling them to overcome challenges related to nonlinear dynamics, hysteresis, and complex control requirements. ML and DL approaches provide powerful data-driven tools for modeling, perception, and control, allowing soft robots to learn optimal behaviors and adapt to changing conditions without relying on predefined analytical models. This synergy between soft robotics and artificial intelligence has led to breakthroughs in autonomous locomotion, manipulation, and environmental interaction, positioning soft robots as promising solutions for a range of applications, from biomedical devices to search and rescue operations [1,2,3].

Despite recent advances, achieving robust and energy-efficient locomotion in soft robots remains a significant challenge, particularly in unstructured environments. This paper aims to address this gap by developing a data-driven framework for optimizing the locomotion of a bio-inspired soft inchworm robot, enabling adaptive and efficient movement across diverse terrains.

Section 2 provides a comprehensive review of recent advances in soft robotics, with particular emphasis on machine learning, deep learning, and reinforcement learning approaches for modeling, control, and adaptive behavior. Section 3 details the bio-inspired design principles, material selection, and fabrication process of the soft inchworm robot, including the experimental setup and data acquisition methods across different surfaces. Section 4 presents the methodology for developing and evaluating predictive models, focusing on the implementation and performance of a feedforward neural network for velocity prediction, and compares it against traditional and advanced machine learning techniques. Section 5 introduces the energy efficiency optimization framework, describing how the trained neural network surrogate is integrated with particle swarm optimization (PSO) to maximize locomotion efficiency and minimize energy consumption through surface-specific and adaptive strategies. Section 6 discusses the experimental results, analyzing the improvements in locomotion efficiency, adaptability, and energy savings achieved by the proposed framework across various terrains, and validating the effectiveness of the optimization approach. Finally, Section 7 concludes the paper by summarizing the main findings and outlining future research directions for enhancing adaptive and energy-efficient soft robotic locomotion.

## 2. Related Work

Recent developments in deep learning including supervised, reinforcement, and unsupervised learning have significantly advanced the capabilities of soft robotics, especially in areas such as control, perception, and adaptive behavior. These methodologies are particularly impactful for bio-inspired robots, manipulators, grippers, and sensor systems, enabling them to operate effectively in unpredictable and dynamic environments. The integration of artificial, embodied, and mechanical intelligence has fostered three principal adaptive strategies: adaptive shape, adaptive functionality, and adaptive mechanics. These strategies empower soft robots to respond dynamically to environmental changes, leveraging material properties and structural design to reduce reliance on centralized control architectures [4,5,6]. Machine learning techniques play a pivotal role in soft robotics by addressing the inherent challenges of nonlinear behaviors, hysteresis effects, and complex dynamics present in both sensors and actuators [7]. Machine learning, particularly reinforcement learning, has proven especially effective for soft robots such as snake-like and crawling platforms, enabling them to learn optimal locomotion strategies through direct interaction rather than relying on explicit mathematical models. Hybrid approaches, combining physics-based models with deep learning, further enhance the modeling of nonlinear behaviors, hysteresis, and complex dynamics in soft actuators and sensors, supporting robust sim-to-real transfer and adaptive control. Advanced neural architectures, including recurrent neural networks and domain randomization techniques, have improved proprioception, environmental interaction, and real-time adaptation. These innovations have resulted in soft robots that can autonomously optimize their movement, adapt to diverse terrains, and maintain high energy efficiency, as demonstrated in recent works on inchworm-inspired and snake-like robots. Despite these advances, challenges remain, including the need for large, unbiased datasets and the ongoing pursuit of robust, energy-efficient locomotion across variable environments [8,9].

Reinforcement learning (RL) has emerged as a highly effective approach for enabling soft robots such as planar snake robots and soft-legged platforms to autonomously adapt their locomotion strategies in complex and unstructured environments without the need for precise mathematical models. RL algorithms allow soft robots to learn optimal movement patterns and control policies directly through interaction with their environment, overcoming the challenges posed by nonlinear dynamics, material hysteresis, and high-dimensional state spaces that are typical in soft robotic systems. For instance, RL-based controllers have been successfully used to optimize gait parameters and motion planning for snake-like robots, facilitating smooth and adaptive navigation even in multi-obstacle scenarios [10,11,12,13]. Recent studies have demonstrated that advanced RL techniques, such as deep Q-learning, actor-critic methods, and proximal policy optimization (PPO), can efficiently train soft robots in simulation and transfer learned policies to real-world hardware, achieving robust performance and real-time adaptability [14]. Furthermore, RL frameworks can be integrated with sensory feedback and domain randomization strategies to enhance sim-to-real transfer, enabling soft robots to perform agile maneuvers and maintain stability across varying terrains and operational conditions [15]. These advancements highlight RL’s transformative potential in soft robotics, supporting the development of autonomous, energy-efficient, and resilient soft robotic systems capable of tackling tasks that are challenging for traditional rigid robots.

Bio-inspired and data-driven optimization and control strategies have significantly advanced soft robot performance. Improved particle swarm optimization (PSO) algorithms and adaptive self-growth strategies enable efficient path planning and obstacle avoidance, while model reference predictive adaptive control (MRPAC) frameworks enhance online adaptation and trajectory tracking in large-scale soft robots. Reinforcement learning (RL) empowers biomimetic soft robots, such as fish- and inchworm-inspired designs, to learn optimal locomotion and navigation behaviors directly on hardware, with proximal policy optimization (PPO) demonstrating superior real-time control among tested RL algorithms. These approaches collectively address the challenges of nonlinearity, dynamic uncertainty, and energy efficiency in soft robotic systems [16,17,18]. Machine learning techniques, particularly neural networks and reinforcement learning, have become central to soft robotic sensing and control, enabling accurate modeling, state estimation, and adaptive control in systems with highly nonlinear and high-dimensional dynamics. These data-driven approaches are especially effective for addressing challenges such as hysteresis, non-stationarity, and variability in soft sensors and actuators, supporting both simulation-based and real-world implementations in soft manipulators and end effectors [19]. Building on this foundation, recent work demonstrates that deep reinforcement learning combined with efficient mechanical modeling, such as Cosserat rod dynamics and domain randomization, enables dexterous soft robotic arms to perform complex dynamic tasks like closed-loop pose and force-controlled pushing. This approach achieves robust sim-to-real transfer and emergent adaptive behaviors, highlighting the potential of learning-based controllers for physical interaction tasks in unstructured environments [20]. Hybrid physics–machine learning frameworks are increasingly used to address the modeling and control challenges of soft robots. For instance, modular soft robotic snakes now leverage proprioceptive feedback and iterative learning control using onboard curvature sensors to autonomously correct gaits and enable robust locomotion and trajectory tracking, while adaptive bounding box motion planning algorithms facilitate efficient navigation and obstacle avoidance in cluttered environments [21]. Complementing these advances, recent work demonstrates that combining Lagrangian mechanics-based surrogate deep neural networks (DNNs) with error-compensating DNNs yields highly accurate, data-efficient models for nonlinear model predictive control. This hybrid approach improves proprioception, environmental interaction, and adaptive locomotion by capturing both general physical dynamics and unmodeled real-world discrepancies, resulting in a 52% average performance improvement over purely analytical or learned models [22].

Ref. [23] presents a learning-based pipeline that alternates between real-world experiments and differentiable simulation, using gradients for joint system identification and trajectory optimization. Model and control parameters are iteratively updated to minimize the gap between simulated and real robot motion. This process effectively narrows the simulation-to-reality gap and improves real-world performance. A bio-inspired control architecture for soft snake robots integrates a reinforcement learning (RL) module with a central pattern generator (CPG) system based on Matsuoka oscillators, enabling adaptive goal-tracking and the generation of stable, diverse locomotion patterns. The RL module, using proximal policy optimization and an option-critic framework, regulates the CPG inputs for real-time steering and velocity control, achieving efficient, data-driven learning and robust goal-reaching behaviors validated in both simulation and physical robots [24]. To address the challenges of modeling and real-time control for soft robots with high degrees of freedom, recent work has combined finite element analysis (FEA) with artificial neural networks (ANNs), enabling shape memory alloy-actuated manipulators to achieve accurate and efficient movement predictions for new actuator inputs while overcoming FEA’s computational limitations in dynamic locomotion tasks [3]. Robust multimodal indirect sensing for movement and interaction has been realized through a recurrent neural network-based adaptive unscented Kalman filter (RNN-AUKF) architecture, leveraging long short-term memory (LSTM) and gated recurrent units (GRUs) to estimate both proprioceptive states and unknown exteroceptive inputs in pneumatic soft fingers, thereby enhancing the accuracy and resilience of locomotion control under sensor degradation [25]. Furthermore, trunk-like soft manipulators demonstrate that reinforcement learning-driven proxy models, combined with finite element method (FEM)-based inverse models, can generate efficient movement and locomotion strategies without the need for precise analytical modeling, supporting adaptive and agile motion in complex environments [26].

A bio-inspired control framework for soft snake robots integrates reinforcement learning with a central pattern generator (CPG) network based on Matsuoka oscillators, enabling adaptive and robust goal-tracking locomotion by dynamically regulating oscillatory patterns and converting them into pressure commands for pneumatic actuators, thus achieving smooth, efficient movement without reliance on precise mathematical models [27]. The Geltropod, a soft robot inspired by gastropod locomotion, employs a physical reservoir computing approach that leverages the intrinsic properties of soft matter for environmental sensing; this integration of machine learning and soft material dynamics enables advanced tactile discrimination and adaptive locomotion across diverse surfaces, supporting both movement and environmental interaction [28]. The DeepCPG framework embeds CPG into a hierarchical policy architecture within deep reinforcement learning (DRL) frameworks for soft robots. The method leverages the Kuramoto oscillator model to generate smooth motor trajectories and integrates sensory feedback for coordinated locomotion. Using the Twin Delayed Deep Deterministic (TD3) algorithm, the approach enables sample-efficient end-to-end learning of locomotion strategies in high-dimensional sensor spaces, achieving robust sim-to-real transfer without additional fine-tuning [29]. Moreover, deep Q-networks (DQN) integrate geometric mechanics to optimize locomotion gaits for soft snake robots. The method leverages structural symmetries to reduce system complexity, enabling model-free training and adaptive gait learning in both terrestrial and aquatic environments [30]. A data-driven simulation framework integrates Bayesian optimization (BO) and Gaussian processes (GPs) to optimize locomotion strategies for magnetic soft millirobots. The method learns periodic magnetic actuation signals in simulation and transfers them to real-world environments using sim-to-real transfer techniques, enabling adaptive locomotion in unknown environments through automated domain recognition based on Kullback–Leibler divergence [31].

A deep reinforcement learning (DRL) framework was developed for a soft underwater snake robot actuated by dielectric elastomer actuators. This enabled model-free, data-driven training of neural network controllers in simulation and successful transfer to real hardware; the robot could autonomously learn and execute efficient, adaptive swimming gaits in complex aquatic environments without relying on explicit analytical models [32]. Moreover, a soft crawling robot featuring a bioinspired asymmetric sawtooth abdominal surface achieves efficient, adaptive locomotion using a single pneumatic actuator and frictional anisotropy. By dynamically interacting with different substrates through body bending and surface microstructures, the robot generates direction-dependent frictional forces, enabling forward and backward crawling on both flat and rough terrains. Locomotion is autonomously adapted using a sensor-driven neural control system with recurrent neural dynamics and short-term memory, allowing the robot to adjust its gait and navigate confined spaces with minimal actuation and sensing complexity [33]. A bio-inspired perception framework for soft robots integrates embedded soft strain sensors made from carbon nanotube-doped PDMS with long short-term memory (LSTM) neural networks to enable real-time, model-free estimation of both kinematics and external forces during locomotion. This approach allows soft actuators to accurately predict tip position and applied forces, even in the presence of sensor drift, nonlinearities, and partial sensor failure, supporting robust, adaptive movement and closed-loop control in dynamic environments [34]. Moreover, a bio-inspired adaptive neural control system enables soft crawling robots to achieve robust locomotion and dynamic adaptability in complex environments. The system integrates four key modules: a CPG for periodic locomotion, a reservoir computing (RC)-based forward model for sensory prediction, CPG postprocessing, and an online neural learning module with STM [35].

Table 1 provides a comprehensive comparison of major machine learning and deep learning methods applied in soft robotics, highlighting their primary application areas, advantages, and current limitations. By systematically contrasting these approaches, the table clarifies how each method addresses the challenges of nonlinear dynamics, adaptability, and control in soft robotic systems.

Despite significant progress in the application of machine learning and deep learning to soft robotics, key limitations persist that make it difficult to use these systems widely and achieve robust real-world performance. Many state-of-the-art approaches require large, diverse datasets for training, which are often time-consuming to collect and may not generalize well across different environments or robot morphologies, leading to issues with bias and limited adaptability. The inherent nonlinearity, hysteresis, viscoelasticity, and material drift in soft robots further complicate accurate modeling and control, often resulting in performance degradation over time and under varying operational conditions. Additionally, most current methods focus primarily on improving control and perception, frequently overlooking the critical aspect of energy efficiency in locomotion, which is essential for long-term, autonomous operation. The lack of integrated frameworks that can simultaneously address modeling complexity, adaptability, and energy optimization remains a major challenge in the field. Table 2 provides a comprehensive overview of recent advances in data-driven modeling, control strategies, and optimization approaches for bio-inspired soft robots, highlighting key methodologies and their advantages in the field.

Recent advances in soft robotics have leveraged machine learning and optimization techniques to address the challenges of nonlinear dynamics, hysteresis, and complex control in compliant systems. Neural network models, reinforcement learning, and particle swarm optimization (PSO) have each been applied to soft robot locomotion, typically focusing on either improving control accuracy or enabling adaptive behaviors in response to environmental changes. For example, neural networks have been used as surrogate models for kinematic and dynamic prediction, while PSO and other optimization algorithms have been employed to tune gait parameters or plan efficient trajectories. However, most existing approaches treat these techniques independently and often require extensive retraining or large datasets to adapt to new environments. Furthermore, the majority of prior works prioritize control and perception, frequently overlooking the critical aspect of energy efficiency in soft robot locomotion, a key factor for long-term autonomous operation.

To address these gaps, this work introduces a unified, data-driven framework that combines neural network modeling with particle swarm optimization to achieve both adaptive and energy-efficient locomotion in a bio-inspired soft inchworm robot. Distinct from prior studies, our approach adapts neural network modeling by training directly on experimental velocity data collected from the physical robot across diverse surface conditions, ensuring robust generalization without reliance on extensive simulation or analytical models. This surrogate model is then integrated with PSO in an optimization loop to jointly tune actuation frequency and input pressure, targeting maximal velocity-to-pressure ratio and minimal energy consumption for each terrain. By coupling predictive modeling with adaptive optimization, the framework enables the robot to dynamically adjust its locomotion strategy in response to changing environments, achieving notable improvements in energy efficiency and stable movement across tested surfaces. This framework directly integrates experimental data-driven modeling with optimization to enable simultaneous adaptability and energy efficiency in soft robot locomotion, offering a scalable solution for real-world deployment.

## 3. Design Concept and Fabrication

The design and fabrication of the soft inchworm robot are grounded in bio-inspired principles, aiming to replicate the adaptive locomotion strategies of natural organisms, as detailed in our previous work [36]. Key considerations in material selection, actuation architecture, and structural features are briefly summarized here to highlight the essential aspects relevant to this study.

The design problem is formulated around achieving maximal locomotion efficiency and adaptability while minimizing energy consumption. The robot must operate on flat, rigid surfaces with known friction coefficients under stable laboratory conditions to ensure repeatability. Material selection is constrained to thermoplastic polyurethane (TPU 85A) due to its favorable combination of flexibility, durability, and airtightness, which are essential for pneumatic actuation and repeated deformation cycles. The fabrication process uses advanced 3D printing techniques to ensure geometric precision and airtight pneumatic chambers, with critical parameters such as wall thickness, infill density, and flow rate computationally optimized to balance compliance and structural integrity.

The robot’s structure features a 45° sloped tail and precision-engineered contact areas, which synergize with the pneumatic architecture to enhance grip-release efficiency, reduce mechanical complexity, and conserve energy. Lightweight, low-impact velocity sensing is integrated to provide real-time feedback without affecting the robot’s natural dynamics. The experimental platform consists of the 3D-printed robot, a programmable pneumatic actuation system, and a systematic protocol for varying actuation parameters on each surface. The overarching goal is to identify actuation settings (pressure and frequency) that maximize the velocity-to-pressure ratio (locomotion efficiency) and minimize energy consumption for target speeds, ensuring the robot’s adaptive and repeatable performance across all tested terrains. This problem formulation directly informs the selection of materials, structural design, actuation architecture, and experimental methodology, ensuring that each aspect of the robot’s construction supports the demands of adaptive, energy-efficient soft robotic locomotion.

### 3.1. Bioinspired Inchworm Design and Locomotion Mechanism

**Figure 1:**
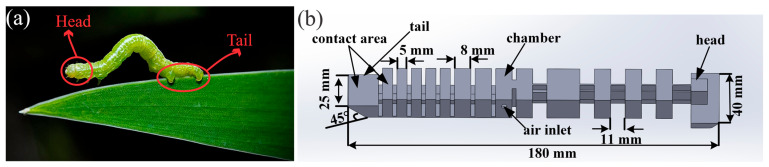
(**a**) Natural inchworm exhibiting peristaltic locomotion. (**b**) The CAD design of the soft inchworm robot including dimensions and details.

The soft inchworm robot’s design emulates natural hydrostatic pressure modulation observed in biological counterparts, where controlled segmental pressure adjustments enable adaptive locomotion across varied terrains. This biomimetic principle drives the robot’s pneumatic actuation system, which achieves bidirectional movement through dynamic bending-angle control via air pressure and actuation frequency modulation (see Figure 1). The inclusion of a 45° sloped tail and precision-engineered contact areas works in concert with the single-tube pneumatic chamber to simplify mechanical complexity, enhance grip-release efficiency, and promote energy conservation. The robot is fabricated from thermoplastic polyurethane (TPU 85A) for its elastomeric properties and compatibility with 3D printing. It integrates a low-friction Teflon air pathway and employs advanced additive manufacturing techniques to ensure both structural integrity and airtightness. Critical design parameters, including wall thickness, infill density, and flow rate, are computationally optimized. This achieves the necessary balance between flexibility, durability, and actuation performance [36].

### 3.2. Data Preparation and Processing

**Figure 2:**
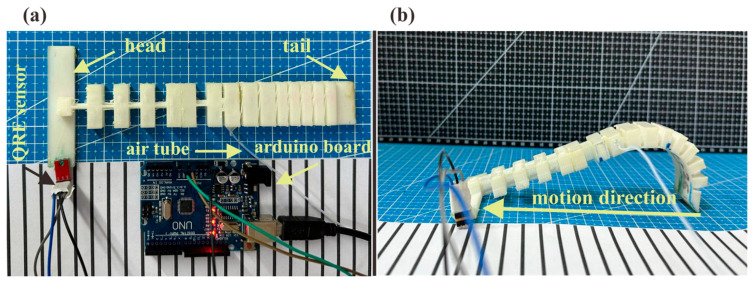
(**a**) Setup of the soft inchworm robot with the sensor and Arduino board. (**b**) The inchworm soft robot under bending condition.

The dataset for training the neural network model was collected from experimental trials of the soft inchworm robot on five different surfaces, each with a specific friction coefficient: glass (GS,μ=2.75), iron (IS,μ=0.27), acrylic (AS,μ=0.84), paper (PS,μ=1.19), and rubber (RS,μ=0.58). A systematic pressure variation protocol was applied, ranging from 50 kPa to 250 kPa in 50 kPa increments and different frequencies, including 1/8, 1/6, 1/4, 1/3, and 2/5 Hz. A QRE1113 reflectance sensor was employed for velocity measurements due to its minimal weight (2 g) and compact size (5 mm × 10 mm), ensuring negligible impact on the robot’s movement. The sensor was mounted on the robot’s head and interfaced with an Arduino Uno board to capture velocity data during each locomotion cycle, which is shown in Figure 2a,b.

### 3.3. Sensor Operation and Velocity Calculation

**Figure 3:**
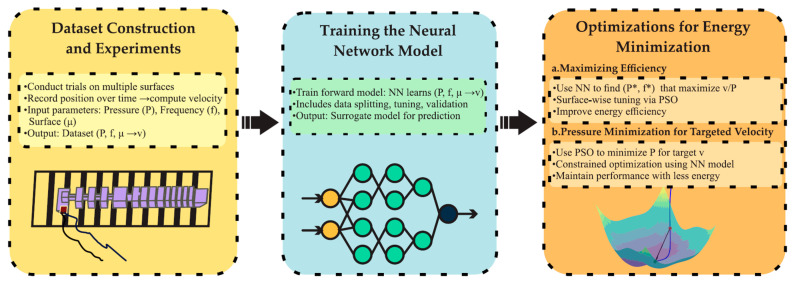
Workflow of the proposed analysis, from experimental data acquisition to model training and energy-efficient actuation optimization.

Motion tracking was implemented using an optical QRE1113 sensor (Phoenix, AZ, USA) paired with a high-contrast patterned surface that detects surface reflectivity using an IR LED and a phototransistor. A black-and-white striped patterned surface was used to generate a digital pulse train corresponding to the robot’s motion. High-reflectivity areas (white strips) produced a strong reflected signal, while low-reflectivity areas (black strips) resulted in a weaker response. The velocity was determined by counting transitions between high (1) and low (0) states, which corresponded to the sensor’s movement over strip boundaries. By tracking the number of these transitions within a given time frame, and considering the width of the strips, the total traveled distance was estimated. The velocity v was computed as:

(1) $$v=\frac{\Delta d}{\Delta t}$$

where Δd=n⋅w represents the cumulative distance traveled, with n denoting the number of detected transitions and w the width of each stripe on the surface. Δt is the corresponding time interval over which these transitions occurred. This approach enabled real-time and accurate estimation of locomotion velocity across various surface materials. The complete procedure for data collection, neural network training, and optimization of the actuation parameters for energy-efficient locomotion is shown in Figure 3.

## 4. Methodology

Soft robotic systems, especially those involving pneumatic actuation and compliant body–environment interactions, exhibit nonlinear and time-dependent behaviors that are difficult to capture using conventional physics-based or analytical models. Factors such as material hysteresis, variable frictional interactions, and actuator dynamics contribute to this complexity. As a result, deriving an explicit mathematical model that maps input parameters (pressure, frequency, and surface material) to output velocity is not feasible or scalable.

Instead, adopting a data-driven modeling approach allows the system to learn underlying patterns directly from experimental observations. This approach supports predictive accuracy, adaptability to varying surface conditions, and seamless integration into subsequent optimization tasks.

### 4.1. Dataset Overview and Evaluation of Traditional Machine Learning Models

The dataset utilized for training comprises 100 data points, each corresponding to a distinct combination of material type, input pressure, actuation frequency, and measured velocity. To enhance the reliability of the model training process, each experimental condition was repeated three times, and the velocity measurements from these trials were averaged. This repetition helps mitigate the impact of random variations, external disturbances, and sensor noise, thereby improving the robustness of the dataset. As a result, the dataset accurately reflects the system’s dynamics, making it more reliable for model training.

To predict velocity from the collected data, several traditional machine learning models were initially explored, including regression-based methods, tree-based models, and instance-based learning approaches. The training process uses the input data (pressure, actuation, and surface friction coefficient) to predict the velocity of the inchworm motion as the model output. Linear regression and lasso regression were initially tested but struggled to capture the nonlinear interactions between the input parameters and velocity. While support vector regression (SVR) offered greater flexibility, it still failed to fully capture the complex feature dependencies, particularly across varying surface materials. Decision tree regression improved performance but was prone to overfitting due to the relatively small dataset size. Ensemble methods, such as random forests and bagging regression, enhanced generalization but still faced limitations in capturing complex relationships within the data.

The performance of all evaluated models to predict the velocity, measured by Mean Absolute Error (MAE), Mean Squared Error (MSE), Root Mean Squared Error (RMSE), and the coefficient of determination (R^2^), is summarized in Table 3. In addition, the specific parameter settings for each machine learning method are detailed in Table 4.

The neural network significantly outperforms the other models, achieving the lowest prediction errors and the highest R^2^ score (0.9362), which corresponds to an overall prediction accuracy of 94.15%, indicating its superior ability to model the complex relationships between actuation parameters and velocity. This performance advantage supports the use of a neural network for velocity prediction.

### 4.2. Predictive Neural Network Framework for Energy-Optimized Locomotion

**Figure 4:**
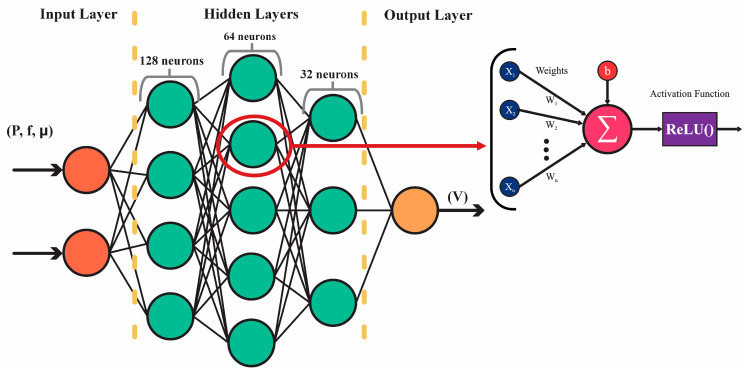
Representation of the feedforward neural network model.

In this study, a feedforward neural network (FNN), as illustrated in Figure 4, is employed to predict the velocity of a soft inchworm robot based on input frequency, actuation pressure, and the surface friction coefficient. These inputs are used to capture the influence of control parameters and environmental interaction on locomotion dynamics. The output of the model is the robot’s resulting velocity. Due to the inherently nonlinear relationships between these parameters, a neural network model is selected for its ability to model complex dependencies with computational efficiency. The neural network architecture comprises three hidden layers with Rectified Linear Unit (ReLU) activation functions, enhancing learning capability and addressing vanishing gradient problems [37]. Dropout regularization is applied to the first hidden layer to reduce overfitting and improve generalization. Six configurations were tested, combining two different numbers of neurons in the first layer (128 and 32) with three dropout rates (0, 0.1, and 0.2). The results indicated that a larger number of neurons in the first layer (128) combined with a dropout rate of 0.1 yielded the best performance, as detailed in Table 5.

The neural network is trained using the Mean Squared Error (MSE) loss function, a common choice for regression tasks. Optimization is performed using the Adam algorithm, which adapts learning rates for each parameter to ensure stable and efficient convergence. The dataset is randomly divided into training (80%) and test (20%) sets.

During training, the model iteratively updates its weights to minimize MSE on the training data, while the test set is used to evaluate generalization. A batch size of 32 and a maximum of 500 epochs were employed, with early stopping based on validation loss to prevent overfitting. The training procedure was repeated with different random seeds to ensure robustness of the results.

The performance of the models is quantitatively evaluated using four standard regression metrics: Mean Absolute Error, Mean Squared Error, Root Mean Squared Error, and the coefficient of determination, as defined in Equations (2)–(5):

(2) $$MAE=\frac{1}{n}\sum_{i=1}^{n}\left|v_{i}-{\hat{v}}_{i}\right|$$

(3) $$MSE=\frac{1}{n}\sum_{i=1}^{n}{\left(v_{i}-{\hat{v}}_{i}\right)}^{2}$$

(4) $$RMSE=\sqrt{\frac{1}{n}\sum_{i=1}^{n}{\left(v_{i}-{\hat{v}}_{i}\right)}^{2}}$$

(5) $$R^{2}=1-\frac{\sum_{i=1}^{n}{\left(v_{i}-\hat{v_{i}}\right)}^{2}}{\sum_{i=1}^{n}{\left(v_{i}-\bar{v}\right)}^{2}}$$

These metrics collectively assess the accuracy and robustness of the neural network predictions. Mean Absolute Error (MAE) and Mean Squared Error (MSE) quantify error magnitude, with MSE penalizing larger deviations more heavily. Root Mean Squared Error (RMSE), provides an interpretable measure in the same unit as the target variable. Coefficient of Determination (R^2^) score indicates the proportion of variance in the target explained by the model, with values closer to 1 denoting better performance.

The final trained neural network reliably captures the nonlinear mapping between actuation inputs and resulting velocity. This predictive capability establishes a foundation for data-driven decision-making in soft robotic control, offering an efficient alternative to physics-based modeling for estimating performance across varying conditions.

## 5. Energy Efficiency Optimization

Energy-efficient locomotion is vital for pressure-driven soft robots navigating diverse terrains. By modulating actuation pressure and frequency in response to surface characteristics, the robot can minimize energy use while maintaining effective movement. The optimization approach builds on neural network predictions to tune these parameters, maximizing velocity output per unit pressure or minimizing pressure for a target velocity, depending on the operational goal and surface friction.

### 5.1. Surface-Specific Energy Optimization via Velocity-to-Pressure Ratio Maximization

Central to this optimization framework is the relationship between input pressure and energy expenditure, where pressure modulation serves as the dominant control variable. By finding the optimal pressure and frequency combination, the system improves its overall energy efficiency, leading to reduced energy usage over time while maintaining effective locomotion. This approach aligns with biomimetic principles observed in natural inchworms, which dynamically adjust internal hydrostatic pressures to balance energy conservation and terrain adaptation. This optimization is performed independently for each surface material, taking into account its specific characteristics, primarily the friction coefficient. For each surface, a predefined range of actuation frequencies $f\in [f_{min},f_{max}]$ and input pressures $P\in \left[P_{min},P_{max}\right]$ is explored using Particle Swarm Optimization (PSO). The trained neural network $\hat{v}(f,P,\mu )$ is used to predict the resulting robot velocity for each candidate combination of f and P, where μ denotes the friction coefficient of the current surface.

To quantify energy efficiency, we define the velocity-to-pressure ratio as follows:

(6) $$\eta \left(f,p,\mu \right)=\frac{\hat{v}(f,P,\mu )}{P}$$

The goal of the optimization is to maximize this efficiency metric η, ensuring that the robot achieves the highest velocity output per unit of input pressure:

(7) $$\left(f*,P*\right)=\arg\underset{f,P}{\max}\left(\frac{\hat{v}\left(f,P,\mu \right)}{P}\right)$$

The Particle Swarm Optimization (PSO) algorithm iteratively searches this design space by initializing a swarm of particles, each representing a candidate solution (f,P). At each iteration, the velocity-to-pressure ratio η is computed for all particles using the neural network prediction. Based on their individual best scores and the swarm’s global best, particles update their velocities and positions to converge toward the optimal solution.

By selecting the optimal pressure and frequency pair (f*,P*) that maximizes the velocity-to-pressure ratio, this optimization process ensures the inchworm-inspired soft robot operates with the highest energy efficiency for each surface condition. This targeted tuning significantly enhances adaptability and reduces energy expenditure, both of which are critical for autonomous soft robotic systems operating in diverse and unpredictable environments. Algorithm 1 shows the pseudocode of the optimization procedure.
**Algorithm 1.** Surface-Specific Energy Optimization via PSO1: **for each** surface material m ∈ M **do**2:          μ←friction coefficient of surface m3:          Initialize swarm with N particles randomly in ($f_{range}\times P_{range}$)4:          **for each** particle i **do**5:                  Predict velocity: ${\hat{v}}_{i}$ ← $NN_{model}(f_{i},\;P_{i},\;\mu )$
6:                  Compute efficiency: $\eta_{i}\;\leftarrow \frac{{\hat{v}}_{i}}{P_{i}}$
7:                  Store personal best: ${p_{best}}_{i}\;\leftarrow \;(f_{i},\;P_{i}),\;{\eta_{pbest}}_{i}\;\leftarrow \;\eta_{i}$
8:          **end for**9:          Set global best gbest ← particle with highest η
10:        **for** $iter\;\in \;[1,\;max_{iter}]$ **do**11:                **for** each particle i **do**12:                        Update velocity and position using PSO rules13:                        Ensure ($f_{i},\;P_{i}$) remain within bounds14:                        Predict velocity: ${\hat{v}}_{i}$ ← $NN_{model}(f_{i},\;P_{i},\;\mu )$
15:                        Compute efficiency: $\eta_{i}\;\leftarrow \frac{{\hat{v}}_{i}}{P_{i}}$
16:                        **if** $\eta_{i}\;>{\eta_{pbest}}_{i}$   **then**17:                            Update $pbest_{i}\;\leftarrow \;(f_{i},\;P_{i}),\;{\eta_{pbest}}_{i}\;\leftarrow \;\eta_{i}$
18:                          **end if**19:                **end for**20:                Update gbest ← best among all $pbest_{i}$
21:        **end for**22:        Store optimal (f*, P*) for surface m ← gbest
23: **end for**

### 5.2. Adaptive Pressure Optimization Locomotion via Surrogate-Assisted PSO

To reduce the input pressure required to achieve a target velocity $v_{target}$, an adaptive optimization strategy is employed. While the relationship between pressure P, frequency f, surface friction μ, and resulting velocity v can be approximately linear in certain scenarios, it often exhibits strong nonlinearities and material-dependent variations. These complexities make analytical inverse modeling intractable for general cases. Therefore, a data-driven approach is adopted, using Particle Swarm Optimization (PSO) in conjunction with a trained neural network surrogate model. This model efficiently predicts the system’s response to actuation parameters, enabling optimization without requiring a closed-form analytical solution.

This inverse optimization process is conducted independently for each material and frequency combination, with each surface characterized by a distinct friction coefficient μ. The neural network is used to predict the velocity, $\hat{v}(f,P,\mu )$, for a given input (f,P). The objective is to determine the minimum pressure P that allows the robot to achieve at least the target velocity:

(8) $$\underset{f,P}{\min}P\;subject\;to\;\hat{v}(f,P,\mu )\geq v_{target}$$

To incorporate this constraint into PSO, a penalized cost function is defined:

(9) $$L(f,P)=P+\lambda \cdot max{\left(0,v_{target}-\hat{v}\left(f,P,\mu \right)\right)}^{2}$$

Here, λ is a penalty coefficient that reinforces the velocity constraint. The PSO algorithm minimizes this loss by iteratively adjusting the candidate solutions, thereby balancing the trade-off between reducing pressure and satisfying the required velocity threshold.

By solving this inverse problem, the optimization framework ensures that the inchworm soft robot operates at the lowest feasible pressure required to meet a predefined locomotion speed. This method enhances energy efficiency by preventing over-actuation, aligning the robot’s behavior more closely with biological inchworms that finely tune internal pressure to respond adaptively to external terrain demands. Algorithm 2 describes this adaptive performance procedure.
**Algorithm 2.** Frequency-Wise Adaptive Pressure Optimization via Surrogate-Assisted PSO1: **for each** surface material m ∈ M **do**2:          μ←friction coefficient of surface m3:          **for each** frequency $f\;\in [f_{min},\;f_{max}]$ **do**4:                  Initialize swarm with N particles $\left(pressures\;P_{i}\right)$ in $\left[P_{min},\;P_{max}\right]$
5:                  **for each** particle i **do**6:                          Predict velocity: ${\hat{v}}_{i}$ ← $NN_{model}(f_{i},\;P_{i},\;\mu )$
7:                          Compute cost: $L_{i}\;\leftarrow \;P_{i}+\lambda \;\cdot \max{\left(0,\;v_{target}-{\hat{v}}_{i}\;\right)}^{2}$
8:                          Store personal best: $pbest_{i}\;\leftarrow \;P_{i},\;cos{t_{pbest}}_{i}\;\leftarrow \;L_{i}$
9:                  **end for**10:                **Set** global best gbest ←particle with lowest cost11:                **for** iter ∈[1, max_iter] **do**12:                        **for** each particle i **do**13:                                Update velocity using PSO rule14:                                Ensure $P_{i}$ remains in bounds15:                                Predict velocity: ${\hat{v}}_{i}$ ← $NN_{model}(f_{i},\;P_{i},\;\mu )$
16:                                Compute cost: $L_{i}\;\leftarrow \;P_{i}+\lambda \;\cdot \max{\left(0,\;v_{target}-{\hat{v}}_{i}\;\right)}^{2}$
17:                                **if** $L_{i}<cos{t_{pbest}}_{i}$ **then**18:                                       Update $pbest_{i}\;\leftarrow \;P_{i},\;cos{t_{pbest}}_{i}\;\leftarrow \;L_{i}$
19:                                **end if**20:                        **end for**21:                        Update gbest ← best among all $pbest_{i}$
22:                **end for**23:                Store optimal pressure P*(f, μ) ← gbest for current frequency24:          **end for**25: **end for**

## 6. Result and Discussion

The optimization framework was evaluated across multiple surface materials, each with distinct friction characteristics, to assess its effectiveness in enhancing energy efficiency and locomotion performance.

Variations in efficiency and motion patterns were observed as a function of pressure and frequency, reflecting the complex, surface-dependent dynamics of the soft inchworm robot. Leveraging neural network predictions and Particle Swarm Optimization, the system was able to adapt actuation parameters for each terrain, achieving notable improvements in pressure efficiency and reducing backward movement tendencies. These results validate the model’s predictive capabilities and its applicability for adaptive control in diverse environments, as summarized in Table 6, which outlines the objectives and design of the experimental tests.

### 6.1. Efficiency Analysis Across Materials

The locomotion behavior of the soft inchworm robot and its efficiency varies significantly across different surfaces due to their distinct material properties. On iron, which has the lowest friction coefficient (μ=0.27), the system exhibits minimal dependency on frequency, with efficiency gradually improving as frequency increases. However, a notable jump in efficiency occurs when the applied pressure exceeds approximately 150 kPa. In contrast, on glass (μ=2.25), the surface with the highest friction coefficient, efficiency is highly dependent on frequency. At lower frequencies (0.125–0.16 Hz), variations in pressure have little impact on performance.

However, at higher frequencies (e.g., 0.4 Hz), efficiency significantly improves, transitioning from backward movement to achieving approximately 0.016 cm/kPa. Paper, on the other hand, exhibits the lowest efficiency among all tested surfaces, as the robot struggles to generate sufficient traction due to surface deformation. The highest efficiency on paper is only achieved at the upper limits of both pressure and frequency. Acrylic demonstrates a lower dependency on pressure but shows the highest sensitivity to frequency among all tested surfaces. At low frequencies, even high pressures fail to improve efficiency. However, beyond 0.3 Hz, the system exhibits significant efficiency gains, with peak performance occurring between 190 and 240 kPa. Rubber surfaces present a distinct locomotion pattern, characterized by the highest backward movement efficiency observed at 150 kPa and frequencies above 0.2 Hz. Forward locomotion on rubber is more responsive to pressure changes, with efficiency improving significantly as pressure increases from 200 kPa to 250 kPa.

Overall, the results demonstrate that actuation frequency has a more pronounced effect on locomotion efficiency compared to input pressure, making it a key factor for energy-aware optimization. The nonlinear dynamics observed across various surfaces underscore the need for a predictive model to effectively tune actuation parameters. By utilizing such a model, the robot can adaptively select frequency and pressure values to maximize the velocity-to-pressure (v/P) ratio, ensuring energy-efficient movement tailored to different terrains. The optimization framework successfully increased energy efficiency, as reflected by an average 9.88% reduction in required pressure to achieve the maximum v/P ratio, with the iron surface showing the highest improvement at 18.32%.

**Figure 5:**
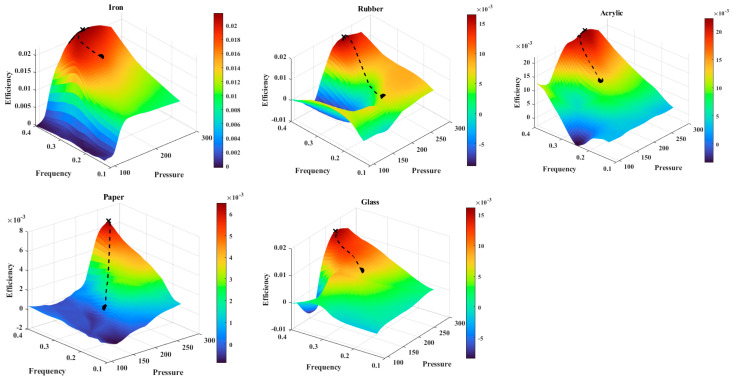
Efficiency surfaces for different materials (iron, rubber, acrylic, paper, and glass). The color intensity represents efficiency values, with higher efficiency shown in warmer colors (red) and lower efficiency in cooler colors (blue). The dashed black line represents the PSO trajectory, which optimizes efficiency by adjusting pressure and frequency. The black dot marks the starting point, and the black cross indicates the optimal solution found by the algorithm.

The model’s predictions align with experimental observations, confirming its ability to identify iron as the most energy-efficient surface and paper as the least efficient. These results further validate the neural network’s capacity to generalize and provide reliable predictions in diverse environmental conditions. Table 7 presents the optimal pressure–frequency combinations that yield the highest efficiency for each tested material. The results highlight the distinct actuation settings required by different surfaces to achieve peak performance. Furthermore, Figure 5 visualizes the efficiency of the inchworm soft robot across various materials, emphasizing the variations in energy consumption and locomotion performance. This figure provides a clear combative analysis of how surface properties influence the effectiveness of the locomotion strategy.

### 6.2. Adaptive Pressure and Frequency Optimization for Material-Specific Locomotion Using PSO

The optimizing of the minimum required pressure to achieve a target velocity reveals significant material-dependent behavior. On iron, the system exhibits negligible backward movement due to the minimal friction resistance [36]. This behavior, captured by the trained neural network, was confirmed through physical experiments, where no backward motion was observed on iron. The neural network model successfully learns that once the robot moves forward on iron, energy loss due to slipping is minimal, enabling operation at lower pressures without performance degradation.

In contrast, glass and paper surfaces exhibit the highest propensity for backward movement. The increased friction on these surfaces helps maintain the robot’s position, preventing undesired slipping and ensuring more stable locomotion. However, this higher resistance necessitates greater input pressures to initiate and sustain forward movement. The trained neural network accurately models these effects, predicting that higher pressures are required to sustain motion on high-friction surfaces like glass and paper compared to lower-friction surfaces like iron. The optimization results, detailing the estimated pressures required for each frequency across different materials, are summarized in Table 8. A key observation from the optimization process is the variation in the robot’s response across different materials and actuation frequencies.

Certain materials exhibit a more linear relationship between pressure and velocity, where increasing pressure leads to predictable and proportional changes in velocity. In contrast, other materials display nonlinear or inconsistent responses, with similar pressure increases yielding varying locomotion performance. The influence of actuation frequency further modulates this behavior, as some materials operate more efficiently within specific frequency bands, while others show diminished or erratic velocity gains. The trained neural network successfully captures these complex interactions, highlighting the critical role of both surface-dependent friction characteristics and frequency selection in determining effective locomotion. Notably, optimization results demonstrate a reduction in the required input pressure for maintaining efficient locomotion, from the 150 kPa baseline reported in our previous study [36], by an average of 6.45%, with a maximum reduction of 25.60% achieved on iron surfaces. This underscores the advantage of leveraging predictive models and optimization techniques to improve energy efficiency across diverse terrains.

The sensitivity of the inchworm soft robot to varying materials and actuation frequencies emphasizes the need for adaptive control strategies. Uniform operating conditions do not consistently yield optimal results across all surfaces. Specifically, some materials exhibit optimal movement at higher frequencies, while others require lower frequencies to avoid instability or backward motion. This variability highlights the necessity of surface-aware and frequency-aware optimization techniques to ensure efficient and stable locomotion across diverse environments. The neural network’s ability to accurately model these complex behaviors reinforces the reliability of the optimization framework, enabling the inchworm robot to dynamically adjust both input pressure and frequency based on surface characteristics. Through this model-driven optimization, the robot can achieve optimal performance with minimal energy consumption, enhancing its adaptability for practical, real-world applications.

## 7. Conclusion and Future Work

This study demonstrates the effectiveness of a data-driven framework that integrates feedforward neural network modeling with particle swarm optimization to achieve energy-efficient and adaptive locomotion in a bio-inspired soft inchworm robot. The FNN accurately captures the nonlinear relationships between actuation parameters (pressure, frequency) and environmental conditions (surface material), outperforming traditional machine learning models in velocity prediction and achieving 94.15% in predicting the inchworm velocity. The two-stage optimization, maximizing the velocity-to-pressure ratio for energy efficiency and minimizing pressure for stable locomotion, yields a 9.88% reduction in energy consumption and a 6.45% reduction in required pressure for stable operation across diverse terrains. This dual approach ensures both energy savings and robust adaptability, enabling real-time, terrain-aware tuning without the need for explicit analytical models. The proposed framework advances the scalability and deployment of soft robots in unstructured environments. Future work will focus on integrating physics-informed neural networks to embed physical constraints directly into the learning process and developing real-time adaptive control strategies to enhance dynamic performance and resilience in complex, changing terrains.

## Figures and Tables

**Table 1:** Comparison of machine learning, deep learning, and neural network techniques in soft robotic systems.

Method/Model	Application Area	Key Advantages	Limitations/Gaps
Linear Regression/Lasso [7]	Simple modeling, baseline comparison	Interpretable, fast	Cannot capture nonlinearities in soft robot dynamics
Decision Tree/Random Forest [4]	Regression, state estimation	Handles nonlinearity, robust to noise	Overfitting, less effective with high-dimensional data
K-Nearest Neighbors (KNN) [7]	Tactile sensing, regression	Non-parametric, easy to implement	Sensitive to noise, not scalable
Support Vector Regression (SVR) [7]	Regression	Good for small, high-dim data	Limited for highly nonlinear, dynamic systems
Feedforward Neural Network (FNN) [4,7]	Kinematic/dynamic modeling	Captures nonlinear relationships, high accuracy	Requires more data, black-box
Convolutional Neural Network (CNN) [4,7]	Tactile/vision-based sensing	Feature extraction from spatial data, robust to noise	Needs large datasets, less suited for time-series
Recurrent Neural Network (RNN), LSTM, GRU [4,5,7]	Dynamic/temporal modeling	Handles time dependencies, memory, hysteresis	Training instability, data-hungry
Deep Reinforcement Learning (DRL) [4,5,7]	Adaptive locomotion, control	Learns optimal policies model-free, adapts to new tasks	Data-intensive, sim-to-real gap, slow training
Hybrid (Physics + ML) [23]	Modeling, sim-to-real transfer	Combines physical priors with data-driven adaptation	Integration complexity, limited scalability
CPG + RL Hybrid [4]	Gait generation, snake robots	Enables rhythmic, adaptive locomotion, reduces RL training	Needs careful tuning, still data-intensive
Reservoir Computing [4]	Tactile discrimination, adaptive control	Leverages soft material dynamics for computation	Limited to specific tasks, less explored in control
Gaussian Processes [23]	Uncertainty estimation, regression	Probabilistic, quantifies uncertainty	Not scalable to large datasets

**Table 2:** Comparative overview of data-driven control and modeling approaches in bio-inspired soft robotics.

Ref.	Robot/Application	Actuation System	Control Method	Modeling Approach	Key Advantage
[1]	Pangasius fish	Servo-driven fin ray	Reinforcement learning (PPO, A2C, DQN tested and PPO)	Model-free RL; direct training on physical robot using pose estimation via DeepLabCut	RL enables the robot to: -Efficiently swim; -Handle complex underwater dynamics without explicit modeling; -Learn goal-reaching behavior on real hardware; -Robust, adaptive control in real aquatic settings.
[2]	Soft robots	Multiple	Machine learning (CNN, kNN, SVM, RNN)	Data-driven (large-scale tactile datasets, sensor fusion)	ML methods effectively: -Handle nonlinearity and hysteresis; -Support versatile sensing and robust interaction in diverse environments.
[3]	Soft bioinspired manipulator	Shape memory alloy	Open-loop (vision-based feedback for validation)	Multilayer Perceptron (MLP) neural network	Neural network enables: -Real-time kinematic modeling; -Reducing the need for extensive physical experiments; Providing fast, energy-efficient direct kinematic solutions.
[4]	Soft robots (review, ML techniques)	Multiple	Machine learning (RNN, hybrid, various)	Data-driven (recurrent/hybrid models)	RNNs and hybrid models: -Capture temporal nonlinearities and dynamics; -Learning-based methods improve real-time modeling; -Control and design optimization in soft robotics.
[6]	Various soft robot	Multiple	Deep reinforcement learning (DRL) and imitation learning (IL) methods including DQN, DDPG, A3C, PPO, GAIL, behavioral cloning, and inverse RL	Model-free (policy/value-based RL, actor-critic, meta-learning), neural networks (CNNs, RNNs), generative models (GANs)	DRL/IL methods enable: -Learning complex, high-DOF soft robot control; -Overcoming the need for explicit analytical models -Meta-learning and imitation learning reduce data requirements and improve real-world deployment;
[8]	Soft robot for laparoscopic surgical operations	Pneumatic actuation	Reinforcement learning (on-policy, proximal policy optimization—PPO)	Neural network (HWB-NN): multilayer perceptron (MLP) with 6D input	-Captures and predicts whole-body morphology including hysteresis effects; -Reduces MSE by 84.95% vs. traditional models -Enables high-precision (0.126–0.250 mm error) real-world trajectory tracking;
[11]	Snake bio-inspired robot	Batteries	Sequence-based deep learning (1D CNN + LSTM), optimized with truncated Newton conjugate-gradient (TNC)	LSTM attention layers, sequence-based model	-Learns efficient adaptive burrowing strategies in highly nonlinear uncertain granular environments.
[12]	Snake soft robot	Motors	Local navigation (RL), gait generation (CPG), gait tracking (PID)	RL (DDPG) tunes CPG parameters for local navigation; CPG generates rhythmic PID tracks joint	-RL-CPG scheme enables fast, transferable learning for high-DOF robots; -Dramatically reduces RL training time.
[13]	Pneumatic soft snake robot	Pneumatic actuation	Model-free reinforcement learning (DDPG, with back-stepping experience replay—BER)	-	-Robust to random target navigation; -Compatible with arbitrary off-policy RL.
[14]	Tripedal soft-legged robot	Tendon-driven	Model-free reinforcement learning (Proximal Policy Optimization, PPO)	Gym environment for RL integration; reward function penalizes instability	-PPO enables learning stable, adaptive walking and navigation without explicit modeling of soft body dynamics; -Achieves 82% success rate in random goal-reaching and 19 mm path deviation on trajectory following;
[15]	Inflated-beam soft robot arm	Pneumatic actuation	Deep reinforcement learning (PPO)	Neural network composed of a multi-layer perceptron (MLP)	-Enables real-time, agile, and highly dynamic maneuvers; -Neural network architecture (MLP + LSTM) allows the policy to leverage temporal information and handle partial observability.
[18]	Soft-rigid hybrid biomimetic fish robot	Servo-driven fin ray	Model-free reinforcement learning (tested PPO, A2C, DQN; PPO)	RL environment built with OpenAI Gym and Stable Baselines3; observation space from real-time pose estimation using DeepLabCut	RL enables the robot to: -Autonomously learn efficient swimming policies for goal-reaching in a real aquatic environment.
[20]	Soft continuum manipulators	Pneumatic actuation	Supervised learning (for sensing, kinematics, dynamics), reinforcement learning (for direct control)	Neural networks (feedforward, convolutional, recurrent/LSTM, deep NN)	ML enables modeling and control of highly nonlinear, high-DOF, and hysteretic soft systems where analytical models are intractable; neural networks can encode dynamic behaviors, adapt to nonstationarity;
[21]	Soft robotic arm	Pneumatic actuation	Deep reinforcement learning (proximal policy optimization—PPO) with closed-loop pose/force control	Dynamic Cosserat rod model with domain randomization in simulation	-Enables sim-to-real transfer for dynamic interaction tasks; -Achieves pose and force control without explicit force input.
[25]	Soft snake robot	Pneumatic actuation	Hybrid: model-free reinforcement learning (PPOC) regulates a central pattern generator (CPG) network (Matsuoka oscillators)	Matsuoka CPG network	-Adaptive RL with stable and diverse CPG-based enables efficient learning in motion.
[26]	Robotic grasping	-	Deep learning-based control (using DNNs)	Deep neural networks (DNNs)	DNNs can learn directly from raw sensor data, extract features without manual engineering, integrate high-dimensional and multimodal data, and adapt to unstructured environments.
[29]	General robotics	-	Deep learning-based control (including reinforcement learning, policy learning, perception-action loops)	Deep neural networks (DNNs), including CNNs, RNNs, autoencoders, and policy networks	DNNs can process raw, high-dimensional sensor data without manual feature engineering, enable sensor fusion, learn complex nonlinear mappings, and adapt to unstructured environments.
[32]	snake robots	Motor-driven joints	Deep reinforcement learning (DQN)	Geometric mechanics-based kinematic models; model-free RL (DQN)	Enables automatic discovery of efficient novel gaits for both land and water without human-designed trajectories.
[34]	Grasping robot	-	Deep learning (various DNN structures	Deep neural networks (DNNs)	DNNs can learn directly from raw sensor data, extract features without manual engineering, fuse multimodal inputs, and adapt to unstructured environments.

**Table 3:** Performance comparison of machine learning models for velocity prediction, sorted by increasing complexity.

Model	MAE	MSE	RMSE	$R^{2}$
Linear Regression	0.6708	0.7385	0.8593	0.6035
Lasso Regression	0.6715	0.7719	0.8786	0.5856
Decision Tree	0.3704	0.3324	0.5765	0.8215
KNN	0.6600	0.9075	0.9527	0.5128
SVR	0.6352	0.8026	0.8959	0.5691
Random Forest	0.4414	0.3387	0.5820	0.8181
Bagging	0.3379	0.3867	0.6219	0.7924
Neural Network	0.2177	0.1520	0.3898	0.9362

**Table 4:** Parameter specifications of machine learning methods.

Method	Parameters
Linear Regression	Intercept: Included Robust: false (ordinary least squares)
Lasso Regression	Lambda (regularization strength): 0.1 Alpha (L1 ratio): 1 (pure Lasso) Intercept: Included Standardize: true
Decision Tree	SplitCriterion: ‘mse’ MaxNumSplits: unlimited MinLeafSize: 1 Prune: false
KNN	Number of Neighbors: 5 Distance: ‘Euclidean’ Weights: ‘uniform’
SVR	Kernel Function: ‘linear’ BoxConstraint (C): 1 Epsilon: 0.1 Standardize: true
Random Forest	Method: ‘Bag’ Number of Learning Cycles: 100 Learner Type: templateTree Feature Subsampling: Yes
Bagging	Number of Learning Cycles: 100 Learner Type: default decision trees Feature Subsampling: No
Neural Network	Input Layer Units: 3 (number of input features) First Layer: Units: (128.32) (Number of neurons in the first layer) Activation Function: ReLU (Rectified Linear Unit) Dropout Layer: Dropout: (0, 0.1, 0.2) Second Layer: Units: 64 (Number of neurons in the second layer) Activation Function: ReLU Third Layer: Units: 32 (Number of neurons in the third layer) Activation Function: ReLU Output Layer Units: 1 (number of outputs) Loss Function: Mean Squared Error (MSE) for regression Optimizer: Adam (Adaptive Moment Estimation) Epochs: 500 Batch Size: 32 Learning Rate: 0.0005 Shuffle: Every Epoch

**Table 5:** Performance comparison of neural network architectures with different configurations.

No.	1st Layer Neurons	Dropout	2nd Layer Neurons	3rd Layer Neurons	MAE	MSE	RMSE	$R^{2}$
1	32	0	64	32	0.2571	0.1692	0.4113	0.9289
2	32	0.1	64	32	0.2801	0.2277	0.4772	0.9044
3	32	0.2	64	32	0.4267	0.4097	0.6401	0.8279
4	128	0	64	32	0.2198	0.1608	0.401	0.9325
5	128	0.1	64	32	0.2177	0.1520	0.3898	0.9362
6	128	0.2	64	32	0.2218	0.1600	0.4000	0.9328

**Table 6:** Summary of experimental tests and objectives.

Experiment	Objective	Surface Materials	Varied Parameters	Evaluation Metric
Exp. 1	Maximize energy efficiency (velocity-to-pressure ratio)	iron, glass, acrylic, paper, rubber	Pressure, frequency	Maximum v/P ratio
Exp. 2	Minimize required pressure for stable velocity	iron, glass, acrylic, paper, rubber	Pressure (per frequency)	Minimum pressure for stable motion

**Table 7:** Optimal pressure and frequency on each surface material and resulted efficiency.

Material	Optimal Pressure (kPa)	Optimal Frequency (Hz)	Predicted Velocity (cm/s)	Efficiency (cm/kPa)
Iron	204.18	0.4	4.4342	0.0217
Rubber	228.68	0.4	3.7667	0.0165
Acrylic	235.86	0.4	5.2706	0.0223
Paper	250	0.4	1.3856	0.0055
Glass	232.35	0.4	3.7592	0.0162

**Table 8:** Optimization results for pressure minimization across surface materials with 0.2 cm/s velocity threshold.

Frequency		0.125	0.17	0.25	0.33	0.4
Iron	FMRMP	113	113	111	108	113
	BMRMP	--	--	--	--	--
Rubber	FMRMP	169	103	191	102	168
	BMRMP	147	--	180	119	104
Acrylic	FMRMP	100	100	118	138	109
	BMRMP	--	--	--	--	--
Paper	FMRMP	231	201	182	194	219
	BMRMP	127	145	147	140	154
Glass	FMRMP	231	165	129	100	--
	BMRMP	100	100	100	--	110

FMRMP: forward movement required minimum pressure; BMRMP: backward movement required minimum pressure.

## Data Availability

The data are contained within this article.
